# Nuclear Pore Complex Protein Mediated Nuclear Localization of Dicer Protein in Human Cells

**DOI:** 10.1371/journal.pone.0023385

**Published:** 2011-08-15

**Authors:** Yoshinari Ando, Yasuhiro Tomaru, Ayako Morinaga, Alexander Maxwell Burroughs, Hideya Kawaji, Atsutaka Kubosaki, Ryuichiro Kimura, Maiko Tagata, Yoko Ino, Hisashi Hirano, Joe Chiba, Harukazu Suzuki, Piero Carninci, Yoshihide Hayashizaki

**Affiliations:** 1 RIKEN Omics Science Center, Yokohama, Kanagawa, Japan; 2 Department of Biological Science and Technology, Tokyo University of Science, Noda, Chiba, Japan; 3 Supramolecular Biology, International Graduate School of Arts and Sciences, Yokohama City University, Yokohama, Kanagawa, Japan; George Mason University, United States of America

## Abstract

Human DICER1 protein cleaves double-stranded RNA into small sizes, a crucial step in production of single-stranded RNAs which are mediating factors of cytoplasmic RNA interference. Here, we clearly demonstrate that human DICER1 protein localizes not only to the cytoplasm but also to the nucleoplasm. We also find that human DICER1 protein associates with the NUP153 protein, one component of the nuclear pore complex. This association is detected predominantly in the cytoplasm but is also clearly distinguishable at the nuclear periphery. Additional characterization of the NUP153-DICER1 association suggests NUP153 plays a crucial role in the nuclear localization of the DICER1 protein.

## Introduction

MicroRNA (miRNA) and small interfering RNA (siRNA) are small RNA approximately ∼23 nucleotides in length which influence gene expression through post-transcriptional regulation of complementary target mRNA in the cytoplasm [1]. miRNA has also been linked to transcriptional silencing and heterochromatin formation in the nucleus [2] though the mechanistic details of these processes remain unclear, particularly in mammals.

DICER, widely conserved across eukaryotic lineages, is a member of the RNase III family of endoribonucleases and targets precursor miRNA (pre-miRNA) or long double-stranded RNA (dsRNA) to produce miRNA or siRNA as part of its essential role in various RNA interference (RNAi) pathways [3], [4]. In mammals, the fundamental role of DICER in the RNAi pathway is thought to explain its linkage to a wide range of developmental processes including early development [5], centromeric silencing in embryonic stem (ES) cells [6], oocyte maturation [7], [8], stem cell proliferation [9], and differentiation of many tissues [10], [11], [12].

The *Schizosaccharomyces pombe* DICER1 ortholog Dcr1 primarily accumulates in the nucleus and is associated with the nuclear pore complex at the nuclear periphery [13]. In the nucleus, Dcr1 associates with chromatin independent of the local level of transcriptional activity [14]. In humans, however, the initial discovery linking DICER1 to cytoplasmic RNAi and the subsequent detailed characterization of its functional role in this pathway [15], [16], [17] has led to the prevailing notion that the DICER1 protein is present solely in the cytoplasm [18], [19], [20]. However, several recent lines of investigation have questioned this assumption. First, evidence linking core RNAi components to heterochromatin formation in mammals have been provided by several reports [6], [21]. Second, it has been shown that Dicer-deficient mouse embryonic stem (ES) cells are defective in the maintenance of centromeric heterochromatin structure and centromeric silencing [6]. Third, the DICER1 protein is known to regulate the transcription of an intergenic region of the human and chicken β-globin gene cluster [22], [23]. Finally, human DICER1 associates with ribosomal DNA chromatin on the mitotic chromosomes [24]. Combination of the above observations suggested to us that human DICER1 protein might also localize and function in the nucleus.

Most nuclear proteins are transported into the nucleus through the nuclear pore complex (NPC), a structure comprised of ∼30 different proteins known as nucleoporins (NUPs) which functions as a nuclear “gate” regulating the transport of macromolecules like proteins and nucleic acids across the nuclear membrane [25], [26], via interaction with importin family proteins which often recognize specific amino acid sequences in the imported protein known as Nuclear Localization Signals (NLS). The importin-α family of nucleocytoplasmic shuttling proteins bind with NLS-containing proteins and transport the proteins into the nucleus with the assistance of an importin-β family protein [27]. Some proteins are shuttled independent of importin-α, relying exclusively on importin-β. For example, the importin-β family protein, transportin-1 (TNPO1) binds with proteins containing dsRNA-binding domains (dsRBDs) and transports these proteins into the nucleus [28]. Interestingly, several NUPs of the NPC, long thought to act as passive structural components, were recently reported to have active transporter-like roles involving the binding of nucleus-targeted proteins and the shuttling of these proteins to the NPC for subsequent transport across the membrane [29], [30], [31], [32]. This NUP-based transport is representative of several recent reports describing importin-independent nuclear transport pathways [27], [33], [34], [35]. Given that human DICER1 appears to lack a canonical NLS for nuclear localization, we further reasoned that nuclear transport could be mediated by such non-canonical transport mechanisms that are just beginning to be understood.

We demonstrate here that human DICER1 protein is localized mainly in the cytoplasm but is also clearly present in the nucleoplasm. Further, we find that human DICER1 protein associates with the NUP153 protein in the cytoplasm and also at the nuclear periphery. On the basis of our results, we propose that NUP153 protein assists the DICER1 protein during transport and localization to the nucleus.

## Results

### Nuclear localization of human DICER1 protein

**Figure 1:**
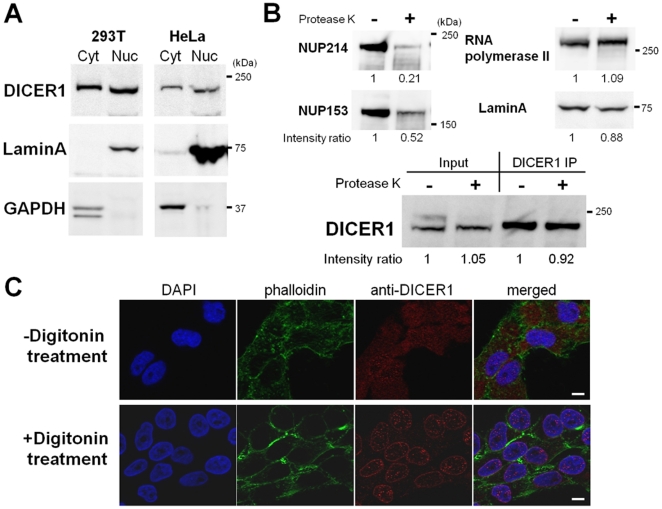
Nuclear localization of human DICER1 protein. (**A**) Western blot analysis for either cytoplasmic (Cyt) or nuclear (Nuc) extracts from 293T and HeLa cells using anti-DICER1, anti-LaminA and anti-GAPDH antibodies. LaminA and GAPDH were used as a nuclear or cytoplasmic marker protein, respectively. Each lane was loaded 50 µg of cytoplasmic extract or 100 µg of nuclear extract, respectively. (**B**) Western blot analysis for isolated nucleus with (+) or without (−) protease K treatment using anti-NUP214, anti-NUP153, anti-RNA polymerase II, anti-LaminA and anti-DICER1 antibodies. The signal intensity of each band was quantified using ImageJ software and intensity ratios were calculated from the “+” sample relative to the “−” sample. “Input” means the sample on 5% of volume used for immunoprecipitation (IP). (**C**) Confocal immunofluorescence images of DICER1 protein in HeLa cells without or with digitonin treatment. The signals of DICER1 protein (red) were detected using anti-DICER1 (12B5/4C6) antibody. Nuclei were counterstained with DAPI (blue) and cytoplasmic regions were co-stained with phalloidin (green). Scale bar represents 10 µm.

To investigate the possibility of nuclear localization of human DICER1 protein, Western blot analysis was performed using the cytoplasmic and nuclear extracts fractionated from 293T and HeLa cells (**Fig. 1A**). Distinctive DICER1 bands were detected on the lanes loaded not only in the cytoplasmic extract but also the nuclear extract. To determine if DICER1 protein was actually present inside the nucleus instead of being present at the surface of the nuclear membrane, we treated isolated nuclei from 293T cells with protease K and performed a Western blot analysis (**Fig. 1B**). The signals of NUP214 and NUP153 proteins, located on the periphery of nuclear pore complex, decreased after treatment while the signals of the RNA polymerase II and LaminA proteins, located in the nucleus, remained about the same. In this condition, the signal of DICER1 protein did not change after protease K treatment (**Fig. 1B**, input). These results were confirmed by immunoprecipitation of the same samples using the anti-DICER1 antibody (**Fig. 1B**, DICER1 IP). These results showed that human DICER1 protein localizes to the inside of the nucleus.

To further confirm the localization of DICER1 protein in the human cells, HeLa cells were immunostained with anti-DICER1 antibody. The confocal image in **Figure 1C** **(−digitonin treatment)** showed that most DICER1 protein signals, shown as red dots, were located in the cytoplasm but several signals overlapped with DAPI staining (blue colored). It was difficult to distinguish whether these signals were in the nucleoplasm or on the surface of the nucleus. Therefore, we permeabilized HeLa cells by digitonin treatment, washed out the cytoplasm and followed by immunofluorescence analysis using anti-DICER1 antibody (**Fig. 1C** **, +digitonin treatment**). Treatment of digitonin in appropriate concentration to the cells increases the permeability of the plasma membrane to cytoplasmic proteins without causing permeabilization of the nuclear membrane. The confocal image showed that DICER1 protein signals remained in the nucleus after digitonin treatment (**Fig. 1C** **, +digitonin treatment**). This supports localization of the DICER1 protein to the nucleoplasm, consistent with the result in **Figure 1B**. Our data demonstrated that human DICER1 protein is located in both the cytoplasm and nucleoplasm.

### Identification of nucleoporins as DICER1-associated proteins

**Figure 2:**
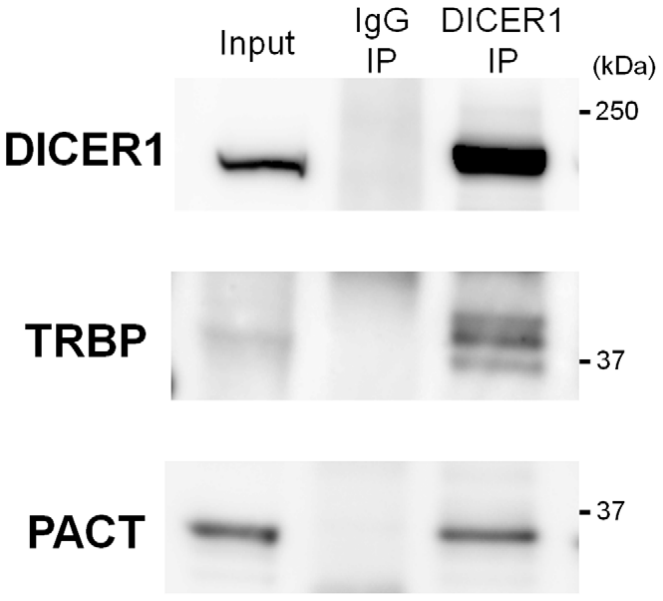
Co-immunoprecipitation (co-IP) of known DICER1-associated proteins with DICER1 protein in HeLa cells. Co-IP experiments using anti-DICER1 (12B5/4C6) antibody from HeLa total cell extracts followed by Western blot analysis with indicated antibodies. “Input” means the sample on 5% of volume used for IP.

As human DICER1 protein lacks a canonical NLS for nuclear localization via interaction of importin-α proteins, this suggested nuclear DICER1 protein could be imported by a non-canonical transport system. In order to identify novel nuclear transport factors associated with human DICER1 protein, we co-immunoprecipitated DICER1-associated proteins using anti-DICER1 antibody from the cytoplasmic extract of 293T cells transiently expressing His-DICER1. TARBP2 (TRBP) [36], [37] and PRKRA (PACT) [38] proteins, known as DICER1-associated proteins, co-immunoprecipitated with DICER1 protein (**Fig. 2**). The proteins were compared with the co-immunoprecipitated proteins from native 293T cells using the same antibody and the changed bands were analyzed using mass spectrometry (MS) (**Table S1**). We could detect four known DICER1-associated proteins (AGO2 [39], KHSRP [40], FMR1 [41] and TRBP [36], [37]) (**Table 1**) as well as several interesting RNA-binding proteins like PUM1 and PUM2 [42], [43], but failed to detect PACT and any importin family proteins in the MS results (**Table S1**).

**Table 1:** Proteins associated with human DICER1 protein.

Gene ID	Gene name	Synonym	Mw	Number of identified peptides	Mascot Score
**Known components of pre-miRNA processing complex**					
23405	DICER1	Endoribonuclease Dicer	217,490	88	2,223
27161	EIF2C2 (AGO2)	Protein argonaute-2	97,146	4	18
8570	KHSRP (KSRP)	Far upstream element-binding protein 2	73,101	2	43
2332	FMR1	Fragile X mental retardation 1 protein	71,131	3	41
6895	TARBP2 (TRBP)	RISC-loading complex subunit TARBP2	39,015	5	85
**Nuclear pore complex proteins**					
8021	NUP214	Nuclear pore complex protein Nup214	213,488	7	78
9972	NUP153	Nuclear pore complex protein Nup153	153,843	35	650
4928	NUP98	Nuclear pore complex protein Nup98-Nup96	187,673	3	29
4927	NUP88	Nuclear pore complex protein Nup88	83,489	1	31
6396	SEC13	Protein SEC13 homolog	35,518	2	54

**Figure 3:**
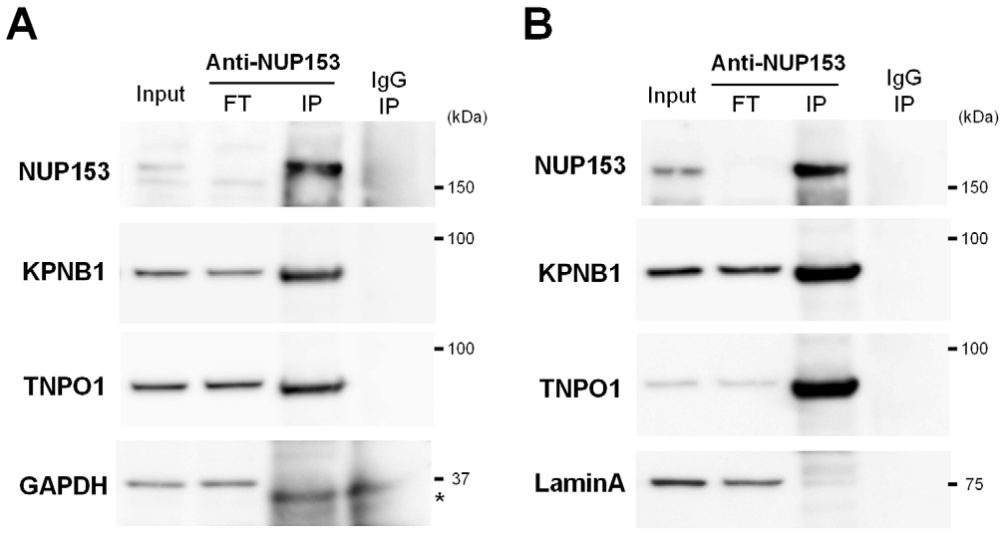
Co-IP of nuclear import receptor proteins with NUP153 protein. (**A**) Co-IP experiments with NUP153 protein from cytoplasmic extracts of HeLa cells followed by Western blot analysis with indicated antibodies. “Input” means the sample on 5% of volume used for IP and “FT” indicates the samples on 5% of flow-through solution of IP samples. The asterisk shows the non-specific band using anti-GAPDH antibody. (**B**) Co-IP with NUP153 protein from nuclear extracts of HeLa cells followed by Western blot analysis with indicated antibodies.

Five NPC proteins (NUP214, NUP153, NUP98, NUP88 and SEC13), previously implicated in nucleocytoplasmic shuttling [29], [30], [31], [32], were detected as candidate interacting proteins (**Table 1**). In particular, the NUP153 protein has been described as a highly mobile nucleoporin [44], [45], [46], [47] which interacts directly with canonical nuclear import factors (**Fig. 3**). We focused our efforts on characterizing the extent of NUP153 protein interaction with the DICER1 protein due to the possibility of the NUP153 protein assisting in nuclear transport and also because the Mascot score [48] of the NUP153 protein was among the highest observed in the MS analysis (**Table 1**).

### DICER1 protein interacts with NUP153 protein in HeLa cells

**Figure 4:**
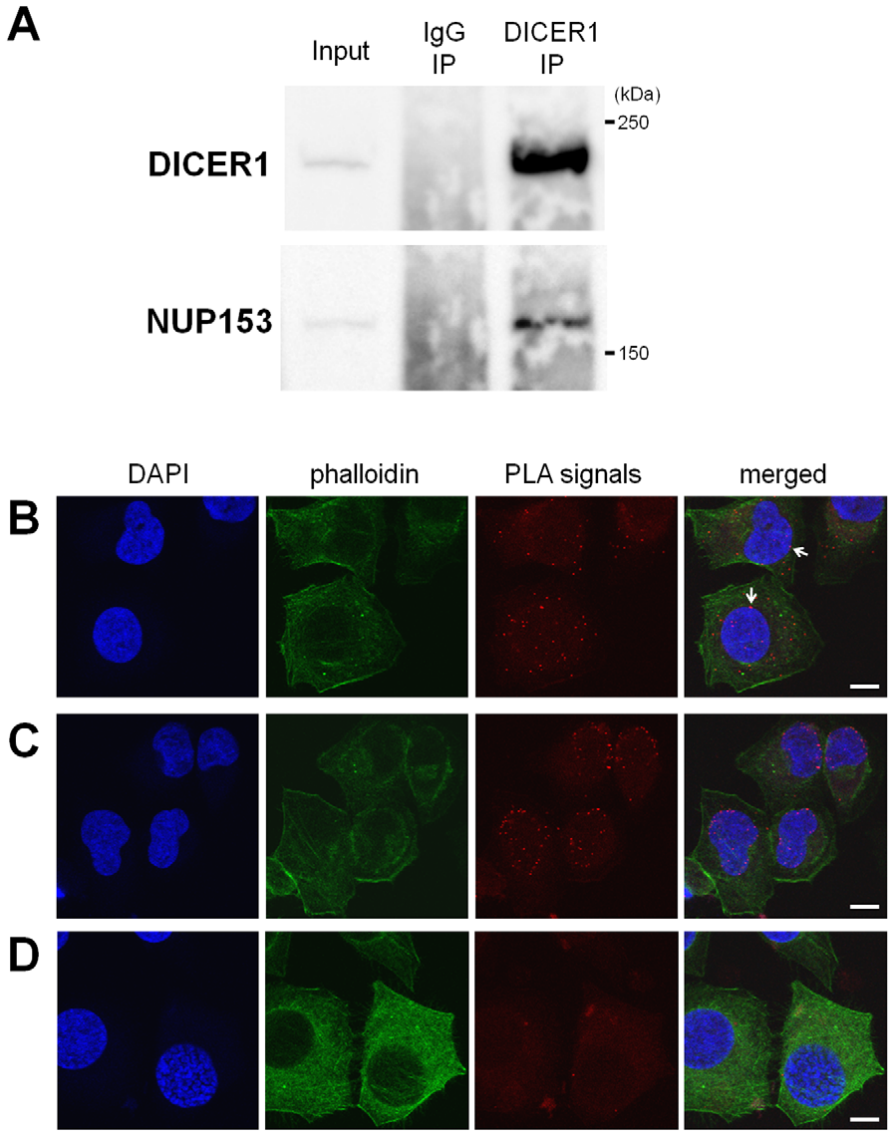
Association of NUP153 protein with DICER1 protein in HeLa cells. (**A**) Co-IP experiments from total cell extracts of HeLa cells followed by Western blot analysis with indicated antibodies. Endogenous NUP153 proteins were immunoprecipitated using anti-DICER1 antibody but not using mouse normal IgG (control). “Input” means the sample on 5% of volume used for IP. (**B**) *In situ* protein-protein associations between DICER1 and NUP153 were detected by Proximity Ligation Assay (PLA). HeLa cells were stained with mouse monoclonal anti-DICER1 and rabbit polyclonal anti-NUP153 antibodies and performed PLA. The association signals were detected by Duolink 100 Detection Kit 613 (red), and nuclei were counterstained with DAPI (blue). Samples co-stained with phalloidin (green) allow visualization of cell borders. Each red dot represents the detection of protein-protein association complex. White arrows indicate the signals at the nuclear periphery. Scale bar represents 10 µm. (**C**) PLA image shows the protein-protein associations between NUP153 and LaminA inside of nuclear membrane. (**D**) A negative control experiment of PLA was performed without addition of any primary antibodies.

To validate the DICER1-NUP153 association, we performed co-immunoprecipitation with anti-DICER1 antibody using whole cell extract from 293T cells. Anti-DICER1 antibody immunoprecipitated with endogenous NUP153 protein, but mouse normal IgG did not (**Fig. 4A**). The co-immunoprecipitation experiments with anti-His antibody were performed using whole cell extract from 293T cells overexpressing His-DICER1 and NUP153 protein was detected in the co-immunoprecipitates (data not shown).

To investigate the association between DICER1 and NUP153 proteins in the cell, an *in situ* Proximity Ligation Assay (PLA) was performed. PLA is a method to detect protein-protein interactions with highly selectivity and sensitivity [49]. Briefly, in PLA, if two modified antibodies binding their respective epitopes are in sufficiently close proximity (typically less than 40 nm), this interaction is detected through emission of a red PLA signal. The PLA signals of DICER1-NUP153 association were detected mainly in the cytoplasm and partly at the nuclear periphery (**Fig. 4B** and **Movie S1**). In contrast, most signals of NUP153-LaminA association were detected only around the nuclear periphery, specifically localizing just inside of the nuclear membrane (**Fig. 4C** and **Movie S2**). No signal was observed in the absence of primary antibodies (**Fig. 4D** and **Movie S3**). This result indicated that DICER1 proteins associate with mobile NUP153 proteins in the cytoplasm, a fraction of DICER1 proteins associated with the NUP153 protein on the periphery of the NPC, and DICER1-NUP153 association was not observed in the nucleoplasm. This suggested that cytoplasmic association with NUP153 protein is meaningful for DICER1 protein and the cytoplasmic NUP153 protein may function in shuttling DICER1 protein to the NPC.

### The NUP153 protein contributes to nuclear import of the DICER1 protein

**Figure 5:**
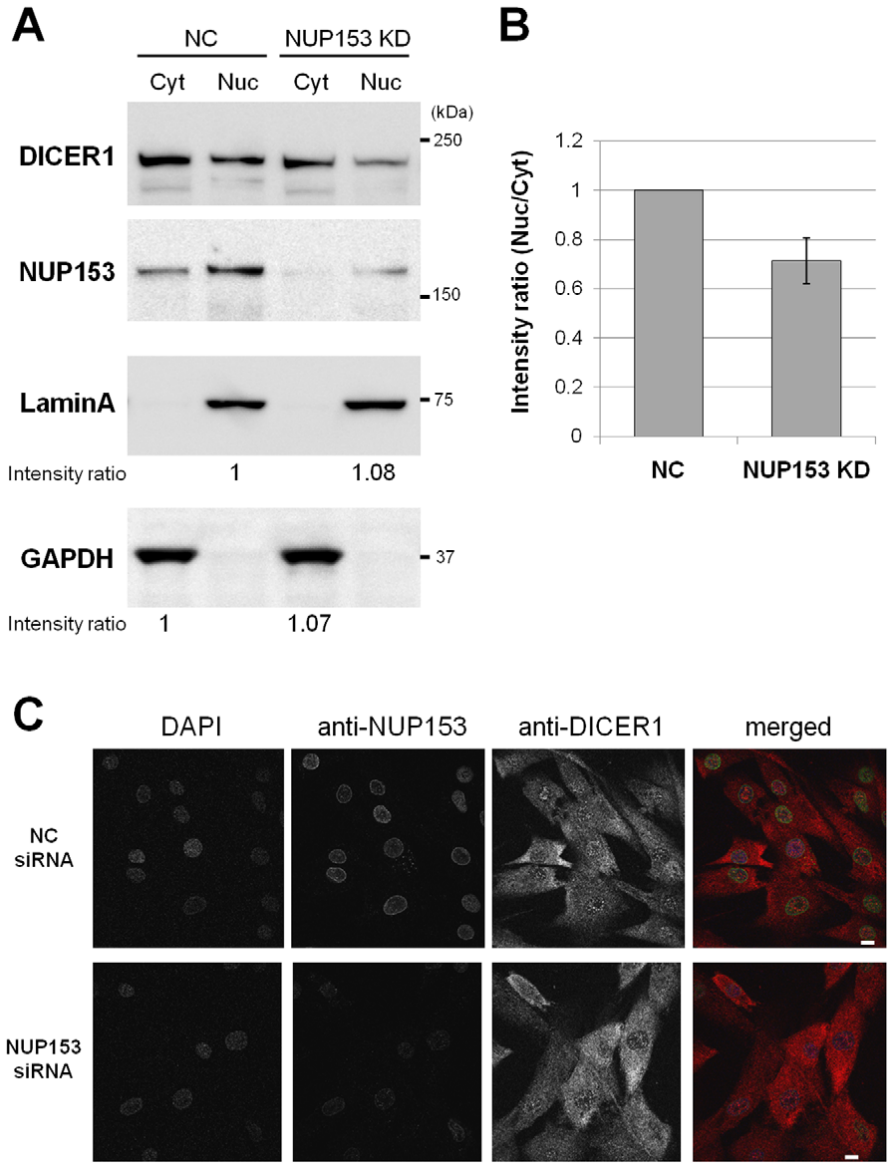
Effects of siRNA knockdown against NUP153. (**A**) Western blot analysis of negative control (NC) and NUP153 knockdown (KD) samples with indicated antibodies. Each lane was loaded 40 µg of cytoplasmic or nuclear extract, respectively. (**B**) Intensity ratio (Nuc/Cyt) of DICER1 protein in NUP153 KD sample is normalized to the intensity ratio of NC sample. The signal intensity of each band was quantified using ImageJ software. These plots show average values of the relative intensity ratio bracketed by s.e.m. error bars; calculated from three independent experiments. (**C**) Confocal immunofluorescence images in human fibroblasts transfected with NC or NUP153 siRNAs. The signals of NUP153 and DICER1 proteins were detected using rabbit polyclonal anti-NUP153 and mouse monoclonal anti-DICER1 antibodies, respectively. Nuclei were counterstained with DAPI. In merged figure, red, green and blue colors represent the signals of NUP153, DICER1 proteins and DAPI, respectively. Scale bar represents 10 µm.

To better characterize the involvement of NUP153 protein in nuclear transport of the DICER1 protein, a knockdown experiment was performed using siRNA for the NUP153 gene. Knockdown efficiency of the NUP153 gene was achieved at an 80% level, as determined by quantitative real-time PCR (qRT-PCR) averaging over three independent experiments (data not shown). This was confirmed by Western blot analysis of HeLa cell extracts using an anti-NUP153 antibody (**Fig. 5A**). The intensity ratio (Nuc/Cyt) of the DICER1 protein was significantly reduced in NUP153 knockdown (KD) samples compared to negative control (NC) samples transfected with NC siRNA (**Fig. 5B**). Meanwhile, the signals of LaminA and GAPDH proteins were not affected by NUP153 KD (**Fig. 5A**). Furthermore, immunofluorescence analysis was performed using human fibroblasts transfected with NC and NUP153 siRNAs (**Fig. 5C**). These results suggest that the NUP153 protein at least partially contributes to DICER1 protein import into the nucleus from the cytoplasm.

A recent report described nuclear import of the human ADAR1 protein via the importin-β-like TNPO1 protein which recognizes and interacts with a dsRBD of ADAR1 [28]. As human DICER1 also contains a dsRBD, we tested the potential role of TNPO1 in possibly supplementing the proposed NUP153-mediated transport. Western blot analysis was performed using co-immunoprecipitated samples with the DICER1 protein. No signal was detected between TNPO1 and DICER1 protein (**Fig. S1**). Interestingly, we similarly tested interaction with the importin-β1 (KPNB1) protein which is linked to importin-α-mediated nuclear transport and detected a very weak signal (**Fig. S1**). This data indicates that while DICER1 is not likely involved in TNPO1-mediated transport, some importin-β family members could contribute to nuclear transport, possibly in conjunction with NUP153.

## Discussion

We demonstrate that DICER1 protein localizes not only to the cytoplasm but, like its counterparts in RNAi, the AGO-like proteins, DICER1 is also found in the nucleoplasm of human cells. This finding has the potential to expand the research fields relating to a small RNA in the nucleus, including its mechanism of biogenesis. In murine cells, pre-mmu-mir-1982 RNA, which is a mirtron with an 11 nt tail at the 5′ end, is spliced out [50]. This unusual pre-miRNA structure is not compatible with nuclear export by Exportin-5 [51]. Despite this, miR-1982* miRNA emerges without 11 nt-5′ overhangs from the deep sequencing data of murine cells [50], [52]. We recently reported that human DICER1 protein could process this pre-mmu-mir-1982 RNA to mature double-stranded miRNA without 5′ overhangs *in vitro* [53]. These observations suggest human DICER1 protein could function in the processing of small RNAs in the nucleus.

Several lines of very recent investigation also hint at other possible function roles for DICER1 in the nucleus. In fission yeast, it was recently reported that Dcr1 protein physically associates with chromatin and H3K9 methylation is not required for the association [14]. Sinkkonen *et al.* showed human DICER1 protein associates with ribosomal DNA loci via immunostaining of mitotic chromosomes [24]. Intriguingly, chromatin immunoprecipitation (ChIP)-seq data with anti-DICER1 (12B5/4C6) antibody suggests the DICER1 protein associates with specific DNA regions and most adjacent genes to the regions were transcribed (unpublished observations, Ando Y, *et al.*). The combination of the above observations together with the experimental data presented in this manuscript could suggest that human DICER1 proteins, while mainly localizing in the cytoplasm as an important component of the RNAi pathway, are also imported actively into the nucleus under the guidance of the NUP153 protein and ultimately associate with active regions of chromatin. Future work will be required to more clearly elucidate functions of human DICER1 protein in the nucleus.

In total, we identified 70 novel DICER1-associated protein candidates from cytoplasmic extract, shown in **Table S1**. In the list, we identified five nucleoporins (NUP214, NUP153, NUP98, NUP88 and SEC13) (**Table 1**). All of these proteins have a demonstrated ability in nucleocytoplasmic shuttling and function in the nucleocytoplasmic transport of macromolecules [29], [30], [31], [32]. Our study links the import of human DICER1 protein with the NUP153 protein. However, it is very likely that another factor also contributes to nuclear import and we cannot rule out the possibility that a decrease of NUP153 protein as a structural component of the NPC may lead to a general decrease in nuclear transport.

We also identified 30 RNA-binding proteins, defined from Gene Ontology (http://geneontology.org/) analysis, and some RISC-associating proteins [54] associating with DICER1 in **Table S1**. Recently, it was reported that two RNA-binding proteins PUM1 and PUM2, identified as DICER1-associated protein candidates in this study, regulate miRNA-dependent gene silencing [42], [43]. The binding of the PUM proteins to target mRNA induces a local conformational change in the 3′ UTR of target mRNA that exposes a specific miRNA-binding site [42]. The DICER1 protein may mediate this regulation via its associations with RNA-binding proteins and RISC-associating proteins.

In summary, these findings have wide-ranging implications for the functional role and interacting partners of human DICER1. We also provide the first possible mode of molecular import via interaction with the nuclear shuttling factor, NUP153.

## Materials and Methods

### Antibodies

Mouse monoclonal anti-hDICER1 (12B5/4C6) was raised in house by using full-length human DICER1 protein as antigens. Rabbit polyclonal anti-hDICER1 (H212, SantaCruz, sc-30226), rabbit polyclonal anti-hTRBP (Abcam, ab42018), rabbit polyclonal anti-hNUP153 (Abcam, ab84872), mouse monoclonal anti-hNUP153 (QE5, Abcam, ab24700), rabbit polyclonal anti-hNUP214 (Abcam, ab70497), mouse monoclonal anti-hLaminA (133A2, Abcam, ab8980), rabbit polyclonal anti-hLaminA (Abcam, ab2559), goat polyclonal anti-hGAPDH (I-19, SantaCruz, sc-48166), mouse monoclonal anti-RNA polymerase II CTD repeat YSPTSPS (4H8, Abcam, ab5408), mouse monoclonal anti-hKPNB1 (31H4, Sigma, I2534) and mouse monoclonal anti-hTNPO1 (D45, Sigma, T0825) were used as primary antibody for Western blotting and immunofluorescence. Mouse Normal IgG (Millipore, 12-371) was used as a control for immunoprecipitation. Alexa flour 488 Donkey anti-rabbit IgG(H+L) (Molecular probe, A11055), Alexa flour 488 Donkey anti-mouse IgG(H+L) (Molecular probe, A21202), Alexa flour 594 Donkey anti-rabbit IgG(H+L) (Molecular probe A21207) and Alexa flour 594 Donkey anti-rabbit IgG(H+L) (Molecular probe A21203) were used as secondary antibody for immunofluorescence. Phalloidin Alexa flour 488 (Molecular probe A12379) and DAPI was used for cytoplasmic and nuclear staining, respectively.

### Cell culture, cytoplasmic and nuclear protein extraction

The 293T and HeLa cells were cultured in DMEM (Invitrogen, 11885) and 10% FBS in a 5% CO_2_ at 37°C. The human normal skin fibroblast cells (NB1RGB), which were established in RIKEN BioResource Center from male 3days old neonate, were cultured in MEM alpha (Wako, 135-15175) supplemented with 10% FBS and Penicillin/Streptomycin (Invitrogen) in a 5% CO_2_ at 37°C. All cell lines were purchased from RIKEN BioResource Center.

Cultured cells were collected, washed twice with cold PBS and incubated in SolutionA (50 mM Tris-HCl pH 7.5, 0.8 M Sucrose, 150 mM Potassium chloride, 5 mM Magnesium chloride, 6 mM β-mercaptoethanol, 0.5% NP-40 and protease inhibitor) for 10 min on ice [55]. Cytoplasmic extracts were cleared by centrifugation at 16,000×g for 15 min at 4°C. Pellets were washed twice with SolutionA (isolated nucleus) and suspended with RIPA buffer by vortexing and sonication. Nuclear extracts were cleared by centrifugation at 16,000×g for 15 min at 4°C. The extracts were quantified using a Protein Assay Kit (Biorad). Isolated nuclei were treated with 2 ng/µl protease K (Invitrogen) in PBS for 5 min at 37°C, washed with PBS and suspended with RIPA buffer by vortexing and sonication.

### Western blot analysis

The protein samples were separated by 4–12% NuPAGE Bis-Tris Gel (Invitrogen) and transferred to the PVDF membrane (Millipore). Detection was achieved with primary antibodies described above and peroxidase-conjugated anti-rabbit (GE Healthcare), anti-mouse (GE Healthcare) and anti-goat (Biorad) antibodies were used as secondary antibodies. The signal intensity of each band was quantified with ImageJ software (http://rsbweb.nih.gov/ij/). The membrane was re-probed by different antibodies after removal of antibodies using Restore PLUS Western Blot Stripping Buffer (Thermo Scientific) from Western blots.

### Cytoplasmic membrane permeabilization

Cells grown on Lab-tek chamber slide (Nunc, 177402) were washed two times with ice cold Transport Buffer (20 mM HEPES pH 7.3, 110 mM Potassium acetate, 5 mM Sodium acetate, 2 mM Magnesium acetate, 1 mM EGTA, 2 mM DTT and protease inhibitor). Washed cells were permeabilized with 40 µg/ml digitonin in Transport buffer for 5 min on ice. Permiabilized cells were preceded to immunoflorescence after washing twice with Transport Buffer and washing twice with PBS [56].

### Immunofluorescence and Proximity Ligation Assay

The procedure for immunofluorescence was essentially as previously described [57] with some modification. Cells grown on Lab-tek chamber slide were fixed with 4% paraformaldehyde in PBS for 10 min at room temperature and permeabilized with 0.5% Triton X-100 in PBS for 4 min at room temperature. The chambers were subsequently incubated for blocking with Blocking One (Nacalai, 03953-95) for 30 min at 37°C. After blocking, cells on chamber were incubated with Blocking One and diluted primary antibody for 45 min at 37°C. After primary antibody incubation, cells were washed with PBS three times and incubated with Blocking One, diluted secondary antibody and phalloidin for 45 min at 37°C. Cells were subsequently washed with PBS three times and mounted with Vectashield with DAPI (Vector laboratories, HT-1200). The Proximity Ligation Assay was performed with DuoLink system (O-link) according to the manufacturer's instructions. Immunofluorescence and proximity ligation assay samples were observed and photographed at 63× magnifications under a confocal laser scanning microscopy system (Leica).

### cDNA cloning and construction of plasmid

We assembled a full-length cDNA of human DICER1 protein from HeLa total RNA. This cDNA sequence was identical to the coding sequence cited in the Swiss-Prot Protein Database (http://au.expasy.org/sprot/) [Swiss-Prot: Q9UPY3]. The cDNA was cloned in a pDEST26 vector (Invitrogen). N-terminally His-tagged human DICER1 protein (His-DICER1) was expressed in 293T cells transfected with the plasmid pDEST26-DICER1.

### Co-immunoprecipitation

Co-immunoprecipitation of DICER1-associated proteins was performed using anti-DICER1 (12B5/4C6) antibody and Dynabeads Protein G (Invitrogen) according to manufacturer's instructions. Each immunoprecipitated protein was detected by Western blot analysis to check for successful co-IP using anti-DICER1, anti-TRBP and anti-PACT antibodies. For the co-immunoprecipitation experiments with NUP153 proteins, anti-NUP153 (QE5) antibody was used. Mouse Normal IgG (Millipore) was used as a control for co-immunoprecipitation. Tenty-five µl of Dynabeads Protein G was mixed with 2.5 µg of the antibody. Then, 200 µg of each cytoplasmic and nuclear extracts in 150 µl B&W buffer (0.1 M sodium phosphate buffer pH 8.2, 0.01% Tween20) was added to the beads-antibody complex and mixed by rotation for 2 hours at 4°C. Supernatants were used as a flowthrough fraction. Beads were washed four times with B&W buffer, and each bound complex was eluted by adding 20 µl of premixed NuPAGE LDS Sample Buffer (Invitrogen) and NuPAGE Sample Reducing Agent (Invitrogen). Immunoprecipitated proteins were separated by NuPAGE Novex 4–12% Bis-Tris gel for Western blot analysis and 10% SDS-PAGE gel for MS analysis.

### Identification of proteins by MS

Protein bands were excised from gels stained by Silver Stain MS Kit (Wako), and in-gel digestion was performed as previously described [58]. Briefly, the gel pieces were washed three times with 60% acetonitrile that contained 50 mM NH_4_HCO_3_, and then dried completely. The dried gel pieces were incubated with 50 mM NH_4_HCO_3_ that contained 25 ng/µl trypsin (Trypsin Gold, MS Grade; Promega) for 16 hours at 37°C. After digestion, 1 µl of formic acid was added to the buffer to stop the reaction. The peptide fragments were desalted and concentrated with ZipTip (Millipore), then eluted with 80% acetonitrile and 0.1% formic acid. The samples were dried completely and solved with 10 µl of formic acid, then injected into LC/LIT-TOF MS (NanoFrontier eLD, Hitachi High-Tech). The peptide mass fingerprints were analyzed using the MASCOT search program (Matrix Science, http://www.matrixscience.com), searching the Swiss-prot database (http://au.expasy.org/sprot/). The quality of peptide product ion spectra is shown as a Mascot score [48].

### siRNA transfection and RNA extraction

Stealth siRNA for NUP153 (5′-UGGGAGUGUUCAGUAUGCUGUGUUU-3′) and NC siRNA (Stealth RNAi Negative Control Medium GC Duplex #2) were purchased from Invitrogen. Transfections of siRNA were performed with Lipofectamin RNAiMAX (Invitorogen) in Opti-MEM medium (Invitrogen) according to the manfacturer's instruction. Total RNAs were extracted 48 hours after transfection with TRIzol (Invitrogen) and FastPure RNA kit (Takara Bio) as previously described [59]. RNA was quantified with NanoDrop (NanoDrop Technologies).

### qRT-PCR for mRNA expression analysis

Expression levels of gene in the gene specific siRNAs or the calibrator negative control siRNA transfected cells were estimated by qRT-PCR with gene specific primer pairs. Reverse transcription reaction was performed with PrimeScript RT-PCR Kit (Perfect Real Time, Takara Bio) according to the manufacturer's instructions. qRT-PCR was performed in 10 µl reaction mixture with SYBR Premix Ex Taq (Perfect Real Time, Takara Bio) on an ABI 7500 Fast Real-Time PCR system (Applied Biosystems). Details of procedure and condition were essentially same as previously described [60]. The primer sequences used for qRT-PCR in this study are NUP153-F: 5′-GGCGACAACAGCATCAGGGCA-3′ and NUP153-R: 5′-TCTGGCCAGCGTGGAACCTC-3′.

## Supporting Information

Figure S1 **Co-immunoprecipitation of nuclear import receptor proteins with DICER1 protein.** Co-immunoprecipitation of DICER1 protein from cytoplasmic extracts of HeLa cells followed by Western blot analysis with indicated antibodies. “Input” means the sample on 5% of volume used for immunoprecipitation (IP) and “FT” indicates the samples on 5% of flow-through solution of IP samples. The asterisk shows the non-specific band using anti-GAPDH antibody.
(TIF)

Movie S1 **Related to** **Figure 4B** **.** Confocal image of PLA using mouse monoclonal anti-DICER1 (12B5/4C6) and rabbit polyclonal anti-NUP153 antibody.
(MOV)

Movie S2 **Related to** **Figure 4C** **.** Confocal image of PLA using mouse monoclonal anti-NUP153 and rabbit polyclonal anti-LaminA antibodies.
(MOV)

Movie S3 **Related to** **Figure 4D** **.** Confocal image of PLA without addition of any primary antibodies.
(MOV)

Table S1 (DOC)
