# Supplementary figures and images for: Nuclear Pore Complex Protein Mediated Nuclear Localization of Dicer Protein in Human Cells
Source: PLoS One. 2011 Aug 15;6(8):e23385. doi: 10.1371/journal.pone.0023385 (PMC3156128; doi:10.1371/journal.pone.0023385)

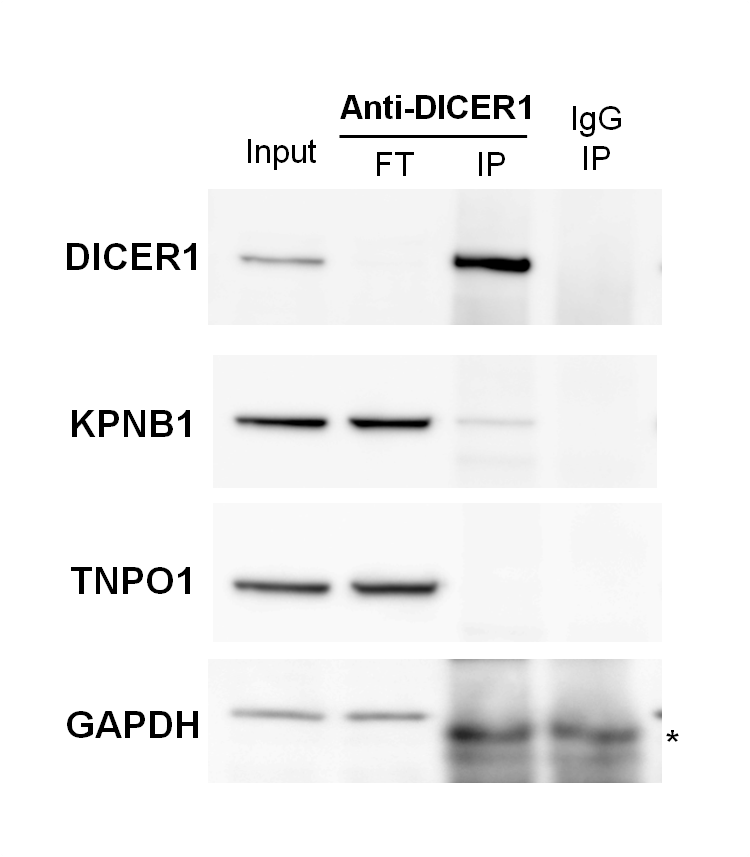

Supplement: Figure S1 — Co-immunoprecipitation of nuclear import receptor proteins with DICER1 protein. Co-immunoprecipitation of DICER1 protein from cytoplasmic extracts of HeLa cells followed by Western blot analysis with indicated antibodies. “Input” means the sample on 5% of volume used for immunoprecipitation (IP) and “FT” indicates the samples on 5% of flow-through solution of IP samples. The asterisk shows the non-specific band using anti-GAPDH antibody. (TIF) [file pone.0023385.s001.tif]
