# Supplementary material for: Nuclear Pore Complex Protein Mediated Nuclear Localization of Dicer Protein in Human Cells
Source: PLoS One. 2011 Aug 15;6(8):e23385. doi: 10.1371/journal.pone.0023385 (PMC3156128; doi:10.1371/journal.pone.0023385)
Supplement: Table S1 — (DOC) [file pone.0023385.s005.doc]

## Supporting Table

**Table S1 Complete list of putative associated proteins with human DICER1 protein detected by MS analysis**

| **Gene ID** | **Gene name** | **Synonym** | **Mw** | **No. of identified peptides / peptide sequence** | **Mascot Score** |
| --- | --- | --- | --- | --- | --- |
| 9919 | SEC16A | Protein transport protein Sec16A | 233,373 | 37 | 496 |
|  |  |  |  | K.FANLTPSR.T |  |
|  |  |  |  | R.FGPGGQLIK.V |  |
|  |  |  |  | K.FATNEAIQR.T |  |
|  |  |  |  | K.KGESWFFR.W |  |
|  |  |  |  | K.DAQGQPGLER.A |  |
|  |  |  |  | R.QSGPGAPNLDR.F |  |
|  |  |  |  | R.YGPLPGPAVPR.H |  |
|  |  |  |  | R.FRELLLYGR.K |  |
|  |  |  |  | R.GLANPEPAPEPK.V |  |
|  |  |  |  | R.TMATMGDTLASR.G |  |
|  |  |  |  | R.TMATMGDTLASR.G + Oxidation (M) |  |
|  |  |  |  | R.TMATMGDTLASR.G + Oxidation (M) |  |
|  |  |  |  | R.QGYPEGYYSSK.S |  |
|  |  |  |  | K.TEAYLPDDKNK.S |  |
|  |  |  |  | R.IVNHWASPELR.Q |  |
|  |  |  |  | R.YVDVLNPSGTQR.S |  |
|  |  |  |  | K.NGLWGHALLLASK.M |  |
|  |  |  |  | R.GGIGASENLENPPK.M |  |
|  |  |  |  | K.LVLIGSNHSLPFLK.F |  |
|  |  |  |  | K.NEHRPASALVNPLAR.G |  |
|  |  |  |  | K.DDTHKVDVINFAQNK.A |  |
|  |  |  |  | R.ADSGPTQPPLSLSPAPETK.R |  |
|  |  |  |  | R.LLPSAPQTLPDGPLASPAR.V |  |
|  |  |  |  | R.AQQELVPPQQQASPPQLPK.A |  |
|  |  |  |  | K.TVQAAPPALPGPPGAPVNMYSR.R |  |
|  |  |  |  | K.TVQAAPPALPGPPGAPVNMYSR.R + Oxidation (M) |  |
|  |  |  |  | K.ANHSSHQEDTYGALDFTLSR.T |  |
|  |  |  |  | K.QIDSSPVGGETDETTVSQNYR.G |  |
|  |  |  |  | R.FANSLPINDPLQTVYQLMSGR.M |  |
|  |  |  |  | K.EKPEEESLAAPTWLVHLQQVER.Q |  |
|  |  |  |  | K.VLSSAASLPGSELPSSRPEGSQGGELSR.C |  |
|  |  |  |  | R.SPDPGIVPQEAPVGNSLSELSEENFDGK.F |  |
|  |  |  |  | R.TLENPVNVYNPSHSDSLASQQSVASHPR.Q |  |
|  |  |  |  | R.GSVSQPSTPSPPKPTGIFQTSANSSFEPVK.S |  |
|  |  |  |  | K.AGSALPGFANSPAGSTSVVLVPPAHGTLVPDGNK.A |  |
|  |  |  |  | R.TVPDSEAPPGWDRADSGPTQPPLSLSPAPETK.R |  |
|  |  |  |  | R.LSGSARPQELVGTFIQQEVGKPEDEASGSFFK.Q |  |
| 7916 | PRRC2A (BAT2) | Large proline-rich protein BAT2 | 228,721 | 14 | 142 |
|  |  |  |  | K.LIPGPLSPVAR.G |  |
|  |  |  |  | R.WGPRPGSSRR.G |  |
|  |  |  |  | R.GGGTGGPNHPPAPR.G |  |
|  |  |  |  | R.SFSSQRPGMER.Q |  |
|  |  |  |  | R.SFSSQRPGMER.Q + Oxidation (M) |  |
|  |  |  |  | K.SLEIQKPAVAPR.H |  |
|  |  |  |  | R.GVGSGGQGPPPPRR.E |  |
|  |  |  |  | K.RSFSSQRPGMER.Q |  |
|  |  |  |  | K.YSSLNLFDTYKGK.S |  |
|  |  |  |  | K.SWAQASVTHGAHGDGGR.A |  |
|  |  |  |  | K.RPPAAPENTPLVPSGVK.S |  |
|  |  |  |  | K.AVGTPGGGGGGAVPGISAMSR.G |  |
|  |  |  |  | K.AVGTPGGGGGGAVPGISAMSR.G + Oxidation (M) |  |
|  |  |  |  | R.LKAEPAAPPAAPSTPAPPPAVPK.E |  |
| 23405 | DICER1 | Endoribonuclease Dicer | 217,490 | 88 | 2,223 |
|  |  |  |  | K.VLPVIK.R |  |
|  |  |  |  | R.LSYMR.S |  |
|  |  |  |  | R.IGIPSTK.Y |  |
|  |  |  |  | K.FSANVPR.S |  |
|  |  |  |  | R.LNLLTPR.H |  |
|  |  |  |  | R.VTVEVVGK.G |  |
|  |  |  |  | K.FMEDIEK.S |  |
|  |  |  |  | K.ALPLSSAEK.R |  |
|  |  |  |  | R.TLLSESPGK.L |  |
|  |  |  |  | R.YTAVVLNR.L |  |
|  |  |  |  | K.TFIAVLLTK.E |  |
|  |  |  |  | R.VTVEVVGKGK.F |  |
|  |  |  |  | K.ALPLSSAEKR.K |  |
|  |  |  |  | K.LYPPEDTTR.C |  |
|  |  |  |  | R.RYTAVVLNR.L |  |
|  |  |  |  | K.VIKLLEILR.K |  |
|  |  |  |  | K.AKWESLQNK.Q |  |
|  |  |  |  | K.AKWESLQNK.Q |  |
|  |  |  |  | R.IGIPSTKYTK.E |  |
|  |  |  |  | R.IGIPSTKYTK.E |  |
|  |  |  |  | R.AQTASDAGVGVR.S |  |
|  |  |  |  | K.AVCLPSILYR.L |  |
|  |  |  |  | R.NKQMEAEFR.K |  |
|  |  |  |  | R.ILGLTASILNGK.C |  |
|  |  |  |  | K.AVCLPSILYR.L + Propionamide (C) |  |
|  |  |  |  | R.ASIVGPPMSCVR.L |  |
|  |  |  |  | R.KLYPPEDTTR.C |  |
|  |  |  |  | R.KLYPPEDTTR.C |  |
|  |  |  |  | R.QHSPGVLTDLR.S |  |
|  |  |  |  | R.YRNFDQPHR.F |  |
|  |  |  |  | R.YRNFDQPHR.F |  |
|  |  |  |  | R.YRNFDQPHR.F |  |
|  |  |  |  | K.SFEEDLKTYK.A |  |
|  |  |  |  | R.KAVCLPSILYR.L |  |
|  |  |  |  | R.SLKANQPQVPNS.- |  |
|  |  |  |  | R.ELLEMEPETAK.F |  |
|  |  |  |  | R.VTINTAIGHINR.Y |  |
|  |  |  |  | K.NEMQGMDSELR.R |  |
|  |  |  |  | R.LPSDPFTHLAPK.C |  |
|  |  |  |  | R.LPSDPFTHLAPK.C |  |
|  |  |  |  | K.FMEDIEKSEAR.I |  |
|  |  |  |  | K.FMEDIEKSEAR.I |  |
|  |  |  |  | K.FMEDIEKSEAR.I + Oxidation (M) |  |
|  |  |  |  | K.NLSVSCAAASVASSR.S |  |
|  |  |  |  | K.LEDYQDAVIIPR.Y |  |
|  |  |  |  | K.IGELDDHLMPVGK.E + Oxidation (M) |  |
|  |  |  |  | K.YIKHEQEELHR.K |  |
|  |  |  |  | K.NLSVSCAAASVASSR.S + Propionamide (C) |  |
|  |  |  |  | R.SSVLKDSEYGCLK.I + Propionamide (C) |  |
|  |  |  |  | K.SNAETATDLVVLDR.Y |  |
|  |  |  |  | R.FIDNMLMGSGAFVK.K |  |
|  |  |  |  | R.MDSEQSPSIGYSSR.T |  |
|  |  |  |  | R.FIDNMLMGSGAFVK.K + Oxidation (M) |  |
|  |  |  |  | R.FIDNMLMGSGAFVK.K + Oxidation (M) |  |
|  |  |  |  | R.MDSEQSPSIGYSSR.T + Oxidation (M) |  |
|  |  |  |  | R.FIDNMLMGSGAFVK.K + 2 Oxidation (M) |  |
|  |  |  |  | R.QQFESVEWYNNR.N |  |
|  |  |  |  | K.ALCPTRENFNSQQK.N |  |
|  |  |  |  | R.FIDNMLMGSGAFVKK.I |  |
|  |  |  |  | R.FIDNMLMGSGAFVKK.I + Oxidation (M) |  |
|  |  |  |  | R.FIDNMLMGSGAFVKK.I + Oxidation (M) |  |
|  |  |  |  | K.HSTIVPENAAHQGANR.T |  |
|  |  |  |  | K.HSTIVPENAAHQGANR.T |  |
|  |  |  |  | R.SPVRELLEMEPETAK.F |  |
|  |  |  |  | R.SPVRELLEMEPETAK.F |  |
|  |  |  |  | R.FYVADVYTDLTPLSK.F |  |
|  |  |  |  | R.SPVRELLEMEPETAK.F + Oxidation (M) |  |
|  |  |  |  | K.GRMDSEQSPSIGYSSR.T |  |
|  |  |  |  | K.GRMDSEQSPSIGYSSR.T |  |
|  |  |  |  | K.FPSPEYETFAEYYK.T |  |
|  |  |  |  | K.GRMDSEQSPSIGYSSR.T + Oxidation (M) |  |
|  |  |  |  | R.TSSLENHDQMSVNCR.T + Propionamide (C) |  |
|  |  |  |  | K.LHKIGELDDHLMPVGK.E |  |
|  |  |  |  | R.TSSLENHDQMSVNCR.T + Oxidation (M); Propionamide (C) |  |
|  |  |  |  | R.APISNYIMLADTDKIK.S + Oxidation (M) |  |
|  |  |  |  | K.ISLSPFSTTDSAYEWK.M |  |
|  |  |  |  | K.ILKSNAETATDLVVLDR.Y |  |
|  |  |  |  | K.KISLSPFSTTDSAYEWK.M |  |
|  |  |  |  | K.KISLSPFSTTDSAYEWK.M |  |
|  |  |  |  | R.ELLEMEPETAKFSPAER.T |  |
|  |  |  |  | R.ELLEMEPETAKFSPAER.T + Oxidation (M) |  |
|  |  |  |  | K.FPSPEYETFAEYYKTK.Y |  |
|  |  |  |  | R.TVFLVNSANQVAQQVSAVR.T |  |
|  |  |  |  | R.TVFLVNSANQVAQQVSAVR.T |  |
|  |  |  |  | K.HLYEDPRQHSPGVLTDLR.S |  |
|  |  |  |  | K.QDPELAYISSNFITGHGIGK.N |  |
|  |  |  |  | K.ISLSPFSTTDSAYEWKMPK.K |  |
|  |  |  |  | K.ISLSPFSTTDSAYEWKMPK.K + Oxidation (M) |  |
| 8021 | NUP214 | Nuclear pore complex protein Nup214 | 213,488 | 7 | 78 |
|  |  |  |  | R.ITPPAAKPGSPQAK.S |  |
|  |  |  |  | R.NRDSGYLHLLYK.R |  |
|  |  |  |  | R.SAQGSSSPVPSMVQK.S |  |
|  |  |  |  | K.TPHPVLTPVAANQAK.Q |  |
|  |  |  |  | K.AAPGPGPSTFSFVPPSK.A |  |
|  |  |  |  | K.TSGVPSGFNFTAPPVLGK.H |  |
|  |  |  |  | K.SHLVHGSSPGVMGTSVATSASK.I |  |
| 23112 | TNRC6B (GW182) | Trinucleotide repeat-containing gene 6B protein | 193,883 | 2 | 38 |
|  |  |  |  | K.GMPFGMGLGNTSR.S |  |
|  |  |  |  | R.AMNLGDFNDIMR.K |  |
| 1981 | EIF4G1 | Eukaryotic translation initiation factor 4 gamma 1 | 175,426 | 2 | 39 |
|  |  |  |  | R.ERPSQPEGLRK.A |  |
|  |  |  |  | R.FSALQQAVPTESTDNRR.V |  |
| 64848 | YTHDC2 | YTH domain containing 2 | 160,147 | 2 | 35 |
|  |  |  |  | R.LSQSLGLVSK.S |  |
|  |  |  |  | R.FHPASVLSQPQYK.K |  |
| 49855 | SCAPER | S phase cyclin A-associated protein in the endoplasmic reticulum | 158,057 | 1 | 32 |
|  |  |  |  | K.VSLATEATR.S |  |
| 90957 | DHX57 | Putative ATP-dependent RNA helicase DHX57 | 155,507 | 3 | 37 |
|  |  |  |  | R.ISAISVAER.V |  |
|  |  |  |  | R.QFQSILQER.Q |  |
|  |  |  |  | K.YSDPPVNFLPVPSR.T |  |
| 9972 | NUP153 | Nuclear pore complex protein Nup153 | 153,843 | 35 | 650 |
|  |  |  |  | K.FSSPIVK.S |  |
|  |  |  |  | K.SPGFASPK.I |  |
|  |  |  |  | R.ILQSLEK.M |  |
|  |  |  |  | K.FGDQGGFK.I |  |
|  |  |  |  | K.MSSPLADAK.R |  |
|  |  |  |  | R.RILQSLEK.M |  |
|  |  |  |  | K.QTGIETPNK.S |  |
|  |  |  |  | K.TTYGGAAAAVR.Q |  |
|  |  |  |  | K.NIVPGWLQR.Y |  |
|  |  |  |  | R.NTPYQAPVRR.Q |  |
|  |  |  |  | K.NVFSSSGTSFSGR.K |  |
|  |  |  |  | R.SGIDITDFQAKR.E |  |
|  |  |  |  | K.TSQLGDSPFYPGK.T |  |
|  |  |  |  | R.LMTPKPVSIATNR.S |  |
|  |  |  |  | R.LMTPKPVSIATNR.S + Oxidation (M) |  |
|  |  |  |  | R.LMTPKPVSIATNR.S + Oxidation (M) |  |
|  |  |  |  | R.EKVDSQYPPVQR.L |  |
|  |  |  |  | R.CHQGPIKPYQQGR.Q |  |
|  |  |  |  | K.FGVSSESKPEEVKK.D |  |
|  |  |  |  | K.QLSAQSYGVTSSTAR.R |  |
|  |  |  |  | R.CHQGPIKPYQQGR.Q + Propionamide (C) |  |
|  |  |  |  | R.IPSIVSSPLNSPLDR.S |  |
|  |  |  |  | R.RCHQGPIKPYQQGR.Q |  |
|  |  |  |  | K.QLSAQSYGVTSSTARR.I |  |
|  |  |  |  | R.SVYFKPSLTPSGEFR.K |  |
|  |  |  |  | R.VTESVKNIVPGWLQR.Y |  |
|  |  |  |  | R.RCHQGPIKPYQQGR.Q + Propionamide (C) |  |
|  |  |  |  | K.EGSVLDILKSPGFASPK.I |  |
|  |  |  |  | R.ILQSLEKMSSPLADAK.R + Oxidation (M) |  |
|  |  |  |  | K.RIPSIVSSPLNSPLDR.S |  |
|  |  |  |  | K.AKQLSAQSYGVTSSTAR.R |  |
|  |  |  |  | R.SVYFKPSLTPSGEFRK.T |  |
|  |  |  |  | R.TEEKQQEPVTSTSLVFGK.K |  |
|  |  |  |  | R.FVASKPLEEEEMEVPVLPK.I |  |
|  |  |  |  | R.FVASKPLEEEEMEVPVLPK.I + Oxidation (M) |  |
| 26058 | GIGYF2 | PERQ amino acid-rich with GYF domain-containing protein 2 | 149,978 | 2 | 33 |
|  |  |  |  | R.ISDQNIIPSVTR.S |  |
|  |  |  |  | R.ALSSGGSITSPPLSPALPK.Y |  |
| 1660 | DHX9 | ATP-dependent RNA helicase A | 140,869 | 14 | 54 |
|  |  |  |  | K.LPIEPR.F |  |
|  |  |  |  | R.LGYIHR.N |  |
|  |  |  |  | K.ILTTEGR.N |  |
|  |  |  |  | R.LNMATLR.M + Oxidation (M) |  |
|  |  |  |  | R.ISAVSVAER.V |  |
|  |  |  |  | R.LNQYFQK.E |  |
|  |  |  |  | R.LGGIGQFLAK.A |  |
|  |  |  |  | R.DFVNYLVR.I |  |
|  |  |  |  | K.LAQFEPSQR.Q |  |
|  |  |  |  | R.ILAKLPIEPR.F |  |
|  |  |  |  | K.YTQVGPDHNR.S |  |
|  |  |  |  | R.VAFERGEEPGK.S |  |
|  |  |  |  | R.TPLHEIALSIK.L |  |
|  |  |  |  | K.LAAQSCALSLVR.Q |  |
| 22907 | DHX30 | Putative ATP-dependent RNA helicase DHX30 | 133,854 | 15 | 230 |
|  |  |  |  | R.ISAVSVAQR.V |  |
|  |  |  |  | R.IPQLLLER.Y |  |
|  |  |  |  | K.NLLNSVIGR.A |  |
|  |  |  |  | R.ALTQFPLPK.N |  |
|  |  |  |  | R.AVAGWEEVLR.W |  |
|  |  |  |  | K.AIFQQPPVGVR.K |  |
|  |  |  |  | R.DLLKEFPQPK.N |  |
|  |  |  |  | R.DLLKEFPQPK.N |  |
|  |  |  |  | R.TPLENLVLQAK.I |  |
|  |  |  |  | R.LQEALGMHESK.Y |  |
|  |  |  |  | R.VSHELGPSLRR.N |  |
|  |  |  |  | R.CQSGFAYHLFPR.S + Propionamide (C) |  |
|  |  |  |  | K.ALLSHDSGSDHLAFVR.A |  |
|  |  |  |  | R.QLNPESIRPGGPGGLSR.S |  |
| 9698 | PUM1 | Pumilio homolog 1 | 126,395 | 5 | 107 |
|  |  |  |  | R.YISAAPGAEAK.Y |  |
|  |  |  |  | R.YGMSDVMPSGR.S |  |
|  |  |  |  | R.DSLTGSSDLYKR.T |  |
|  |  |  |  | K.FWETDESSKDGPK.G |  |
|  |  |  |  | R.SQDDAMVDYFFQR.Q |  |
| 5976 | UPF1 | Regulator of nonsense transcripts 1 | 124,267 | 11 | 131 |
|  |  |  |  | R.LQVQYR.M |  |
|  |  |  |  | R.YGVIIVGNPK.A |  |
|  |  |  |  | K.IPSEQEQLR.A |  |
|  |  |  |  | K.AGLSQSLFER.L |  |
|  |  |  |  | R.FMTTAMYDAR.E |  |
|  |  |  |  | R.EAIIPGSVYDR.S |  |
|  |  |  |  | R.AYQHGGVTGLSQY.- |  |
|  |  |  |  | R.GNTSGSHIVNHLVR.A |  |
|  |  |  |  | K.TVLQRPLSLIQGPPGTGK.T |  |
|  |  |  |  | R.EAIDSPVSFLALHNQIR.N |  |
|  |  |  |  | K.AGAKPDQIGIITPYEGQR.S |  |
| 23196 | FAM120A | Constitutive coactivator of PPAR-gamma-like protein 1 | 121,811 | 6 | 79 |
|  |  |  |  | K.HTPLYER.S |  |
|  |  |  |  | R.GVISTPVIR.T |  |
|  |  |  |  | R.QRPPQTPLR.L |  |
|  |  |  |  | R.QSVLEGLSFSR.Q |  |
|  |  |  |  | R.EAALEAAVLNKEE.- |  |
|  |  |  |  | R.QTAQQIVSHVQNK.G |  |
| 6311 | ATXN2 | Ataxin-2 | 117,044 | 2 | 44 |
|  |  |  |  | R.MGQPGSGSMPSR.S |  |
|  |  |  |  | R.TSPSGGTWSSVVSGVPR.L |  |
| 55833 | UBAP2 | Ubiquitin-associated protein 2 | 117,044 | 3 | 57 |
|  |  |  |  | K.LRESTPGDSPSTVNK.L |  |
|  |  |  |  | K.SQPEPSPVLSQLSQR.Q |  |
|  |  |  |  | K.SQASKPAYGNSPYWTN.- |  |
| 9898 | UBAP2L | Ubiquitin-associated protein 2-like | 114,465 | 10 | 168 |
|  |  |  |  | K.GFGDVGEAK.G |  |
|  |  |  |  | K.QRPQATAEQIR.L |  |
|  |  |  |  | K.GGSTTGSQFLEQFK.T |  |
|  |  |  |  | R.DGSLASNPYSGDLTK.F |  |
|  |  |  |  | K.NPSDSAVHSPFTKR.Q |  |
|  |  |  |  | R.SQTSSIPQKPQTNK.S |  |
|  |  |  |  | R.GNWEQPQNQNQTQHK.Q |  |
|  |  |  |  | R.LAQMISDHNDADFEEK.V |  |
|  |  |  |  | K.SPAVATSTAAPPPPSSPLPSK.S |  |
|  |  |  |  | R.ARGNWEQPQNQNQTQHK.Q |  |
| 23369 | PUM2 | Pumilio homolog 2 | 114,145 | 2 | 42 |
|  |  |  |  | R.FIQQKLER.A |  |
|  |  |  |  | R.YISAAPGAEAK.Y |  |
| 11273 | ATXN2L | Ataxin-2-like protein | 113,304 | 6 | 62 |
|  |  |  |  | K.FGAQFK.L |  |
|  |  |  |  | R.MYPPRSPK.S |  |
|  |  |  |  | K.STSTPTSPGPR.T |  |
|  |  |  |  | R.EIESSPQYR.L |  |
|  |  |  |  | R.AEGLQVGQDAR.V |  |
|  |  |  |  | R.QGSGRESPSLASR.E |  |
| 9967 | THRAP3 | Thyroid hormone receptor-associated protein 3 | 108,601 | 2 | 36 |
|  |  |  |  | R.ASAVSELSPR.E |  |
|  |  |  |  | R.VTAYKAVQEK.S |  |
| 4928 | NUP98 | Nuclear pore complex protein Nup98-Nup96 | 187,673 | 3 | 29 |
|  |  |  |  | K.AGVSTNISTK.H |  |
|  |  |  |  | K.FNPPTGTDTMVK.A |  |
|  |  |  |  | K.VGYYTIPSMDDLAK.I |  |
| 27161 | EIF2C2 (AGO2) | Protein argonaute-2 | 97,146 | 4 | 18 |
|  |  |  |  | K.AVQVHQDTLR.T |  |
|  |  |  |  | K.SGNIPAGTTVDTK.I |  |
|  |  |  |  | K.LTDNQTSTMIR.A |  |
|  |  |  |  | K.NLYTAMPLPIGR.D |  |
| 9782 | MATR3 | Matrin-3 | 94,565 | 5 | 47 |
|  |  |  |  | K.LAEPYGK.I |  |
|  |  |  |  | R.GPGPLQER.S |  |
|  |  |  |  | K.SFQQSSLSR.D |  |
|  |  |  |  | K.FDQKQELGR.V |  |
|  |  |  |  | R.GNLGAGNGNLQGPR.H |  |
| 1665 | DHX15 | Putative pre-mRNA-splicing factor ATP-dependent RNA helicase DHX15 | 90,875 | 2 | 60 |
|  |  |  |  | R.HRLDLGEDYPSGK.K |  |
|  |  |  |  | R.SLMSADNVRQQLSR.I + Oxidation (M) |  |
| 3192 | HNRNPU | Heterogeneous nuclear ribonucleoprotein U | 90,457 | 7 | 94 |
|  |  |  |  | K.DIDIHEVR.I |  |
|  |  |  |  | K.DLPEHAVLK.M |  |
|  |  |  |  | K.NGQDLGVAFK.I |  |
|  |  |  |  | K.LLEQYKEESK.K |  |
|  |  |  |  | R.NFILDQTNVSAAAQR.R |  |
|  |  |  |  | K.SSGPTSLFAVTVAPPGAR.Q |  |
|  |  |  |  | R.NFILDQTNVSAAAQRR.K |  |
| 4927 | NUP88 | Nuclear pore complex protein Nup88 | 83,489 | 1 | 31 |
|  |  |  |  | R.SVANPAFLK.A |  |
| 1653 | DDX1 | ATP-dependent RNA helicase DDX1 | 82,380 | 2 | 35 |
|  |  |  |  | K.APDGYIVK.S |  |
|  |  |  |  | K.ILKGEYAVR.A |  |
| 4076 | CAPRIN1 | Caprin-1 | 78,318 | 5 | 108 |
|  |  |  |  | K.VLKEIVER.V |  |
|  |  |  |  | R.DGYQQNFK.R |  |
|  |  |  |  | R.LNQDQLDAVSK.Y |  |
|  |  |  |  | R.SFMALSQDIQK.T |  |
|  |  |  |  | R.SFMALSQDIQK.T + Oxidation (M) |  |
| 4670 | HNRNPM | Heterogeneous nuclear ribonucleoprotein M | 77,464 | 8 | 119 |
|  |  |  |  | R.MGPGIDR.L |  |
|  |  |  |  | R.MGANSLER.M |  |
|  |  |  |  | R.MGANNLER.M |  |
|  |  |  |  | K.LSGREIDVR.I |  |
|  |  |  |  | K.QGGGGGGGSVPGIER.M |  |
|  |  |  |  | R.MGPAMGPALGAGIER.M |  |
|  |  |  |  | R.MGPAMGPALGAGIER.M + Oxidation (M) |  |
|  |  |  |  | R.MGPLGLDHMASSIER.M |  |
| 22794 | CASC3 | Protein CASC3 | 76,232 | 1 | 30 |
|  |  |  |  | R.AGFRPVEAGGQHGGR.S |  |
| 6421 | SFPQ | Splicing factor, proline- and glutamine-rich | 76,102 | 3 | 58 |
|  |  |  |  | R.AVVIVDDRGR.S |  |
|  |  |  |  | R.GMGPGTPAGYGR.G |  |
|  |  |  |  | R.FGQGGAGPVGGQGPR.G |  |
| 8604 | SLC25A12 | Calcium-binding mitochondrial carrier protein Aralar1 | 74,715 | 1 | 40 |
|  |  |  |  | R.GLIPQLIGVAPEK.A |  |
| 9513 | FXR2 | Fragile X mental retardation syndrome-related protein 2 | 74,178 | 12 | 309 |
|  |  |  |  | R.NLVGKVIGK.N |  |
|  |  |  |  | R.QIPFGDVR.L |  |
|  |  |  |  | R.LQIDEQLR.Q |  |
|  |  |  |  | R.GPPPAPRPTSR.Y |  |
|  |  |  |  | R.ASLLGDMHFR.S |  |
|  |  |  |  | R.ASLLGDMHFR.S + Oxidation (M) |  |
|  |  |  |  | R.IYGETPEACR.Q + Propionamide (C) |  |
|  |  |  |  | R.LRPVNPNPLATK.G |  |
|  |  |  |  | K.VIQEIVDKSGVVR.V |  |
|  |  |  |  | R.IYGETPEACRQAR.S + Propionamide (C) |  |
|  |  |  |  | R.SYLEFSEDSVQVPR.N |  |
|  |  |  |  | K.AGYSTDESSSSSLHATR.T |  |
| 3313 | HSPA9 | Stress-70 protein, mitochondrial | 73,635 | 7 | 155 |
|  |  |  |  | K.VLENAEGAR.T |  |
|  |  |  |  | K.HLNMKLTR.A |  |
|  |  |  |  | K.DAGQISGLNVLR.V |  |
|  |  |  |  | R.TTPSVVAFTADGER.L |  |
|  |  |  |  | K.MKETAENYLGHTAK.N |  |
|  |  |  |  | K.VQQTVQDLFGRAPSK.A |  |
|  |  |  |  | R.QAVTNPNNTFYATKR.L |  |
| 8570 | KHSRP (FUBP2) | Far upstream element-binding protein 2 | 73,101 | 2 | 43 |
|  |  |  |  | K.GGETIKQLQER.A |  |
|  |  |  |  | R.IGGGIDVPVPRHSVGVVIGR.S |  |
| 10521 | DDX17 (p72) | Probable ATP-dependent RNA helicase DDX17 | 72,326 | 28 | 487 |
|  |  |  |  | R.GLDVEDVK.F |  |
|  |  |  |  | K.TIIFVETK.R |  |
|  |  |  |  | R.QLAEDFLR.D |  |
|  |  |  |  | K.STCIYGGAPK.G |  |
|  |  |  |  | K.LMQLVDHR.G |  |
|  |  |  |  | K.LMQLVDHR.G |  |
|  |  |  |  | R.LIDFLESGK.T |  |
|  |  |  |  | K.LMQLVDHR.G + Oxidation (M) |  |
|  |  |  |  | K.LMQLVDHR.G + Oxidation (M) |  |
|  |  |  |  | K.GPQIRDLER.G |  |
|  |  |  |  | K.IVDQIRPDR.Q |  |
|  |  |  |  | K.IVDQIRPDR.Q |  |
|  |  |  |  | R.GVEICIATPGR.L |  |
|  |  |  |  | R.DMVGIAQTGSGK.T + Oxidation (M) |  |
|  |  |  |  | K.APILIATDVASR.G |  |
|  |  |  |  | R.LTPYEVDELR.R |  |
|  |  |  |  | R.KIVDQIRPDR.Q |  |
|  |  |  |  | R.GDGPICLVLAPTR.E |  |
|  |  |  |  | K.VLEEANQAINPK.L |  |
|  |  |  |  | K.NFYVEHPEVAR.L |  |
|  |  |  |  | R.MLDMGFEPQIR.K + 2 Oxidation (M) |  |
|  |  |  |  | R.LTPYEVDELRR.K |  |
|  |  |  |  | R.SSQSSSQQFSGIGR.S |  |
|  |  |  |  | R.SGKAPILIATDVASR.G |  |
|  |  |  |  | R.SGKAPILIATDVASR.G |  |
|  |  |  |  | K.LMQLVDHRGGGGGGGGR.S + Oxidation (M) |  |
|  |  |  |  | R.ELAQQVQQVADDYGK.C |  |
|  |  |  |  | R.ELIKVLEEANQAINPK.L |  |
| 2332 | FMR1 | Fragile X mental retardation 1 protein | 71,131 | 3 | 41 |
|  |  |  |  | R.VGPNAPEEK.K |  |
|  |  |  |  | R.LQIDEQLR.Q |  |
|  |  |  |  | K.LIQEIVDKSGVVR.V |  |
| 10236 | HNRNPR | Heterogeneous nuclear ribonucleoprotein R | 70,899 | 4 | 136 |
|  |  |  |  | K.AGPIWDLR.L |  |
|  |  |  |  | R.TGYTLDVTTGQR.K |  |
|  |  |  |  | R.TGYTLDVTTGQRK.Y |  |
|  |  |  |  | R.DLYEDELVPLFEK.A |  |
| 3312 | HSPA8 | Heat shock cognate 71 kDa protein | 70,854 | 9 | 237 |
|  |  |  |  | K.VCNPIITK.L + Propionamide (C) |  |
|  |  |  |  | R.LSKEDIER.M |  |
|  |  |  |  | K.VQVEYKGETK.S |  |
|  |  |  |  | K.DAGTIAGLNVLR.I |  |
|  |  |  |  | K.NSLESYAFNMK.A |  |
|  |  |  |  | K.NSLESYAFNMK.A + Oxidation (M) |  |
|  |  |  |  | R.ARFEELNADLFR.G |  |
|  |  |  |  | R.TTPSYVAFTDTER.L |  |
|  |  |  |  | K.SFYPEEVSSMVLTK.M |  |
| 8761 | PABPC4 | Polyadenylate-binding protein 4 | 70,738 | 20 | 309 |
|  |  |  |  | K.IIFVGR.A |  |
|  |  |  |  | K.VMLEDGR.S |  |
|  |  |  |  | K.SGVGNVFIK.N |  |
|  |  |  |  | K.EFTNVYIK.N |  |
|  |  |  |  | R.VCRDMITR.R + Propionamide (C) |  |
|  |  |  |  | K.FSPAGPVLSIR.V |  |
|  |  |  |  | K.AKEFTNVYIK.N |  |
|  |  |  |  | K.AKEFTNVYIK.N |  |
|  |  |  |  | R.ALDTMNFDVIK.G |  |
|  |  |  |  | K.EFSPFGSITSAK.V |  |
|  |  |  |  | K.EAAQKVGAVAAATS.- |  |
|  |  |  |  | R.ALDTMNFDVIK.G + Oxidation (M) |  |
|  |  |  |  | R.IMWSQRDPSLR.K |  |
|  |  |  |  | R.KAHLTNQYMQR.V |  |
|  |  |  |  | R.KAHLTNQYMQR.V |  |
|  |  |  |  | R.KAHLTNQYMQR.V |  |
|  |  |  |  | R.KAHLTNQYMQR.V + Oxidation (M) |  |
|  |  |  |  | R.KAHLTNQYMQR.V + Oxidation (M) |  |
|  |  |  |  | R.IVGSKPLYVALAQR.K |  |
|  |  |  |  | K.GFGFVCFSSPEEATK.A |  |
| 26986 | PABPC1 | Polyadenylate-binding protein 1 | 70,626 | 33 | 548 |
|  |  |  |  | K.FGPALSVK.V |  |
|  |  |  |  | K.SGVGNIFIK.N |  |
|  |  |  |  | K.EFTNVYIK.N |  |
|  |  |  |  | K.GFGFVSFER.H |  |
|  |  |  |  | R.KSGVGNIFIK.N |  |
|  |  |  |  | R.VCRDMITR.R + Propionamide (C) |  |
|  |  |  |  | R.YQGVNLYVK.N |  |
|  |  |  |  | K.FSPAGPILSIR.V |  |
|  |  |  |  | K.NLDDGIDDER.L |  |
|  |  |  |  | R.AKEFTNVYIK.N |  |
|  |  |  |  | R.AKEFTNVYIK.N |  |
|  |  |  |  | R.ALDTMNFDVIK.G |  |
|  |  |  |  | R.ALDTMNFDVIK.G + Oxidation (M) |  |
|  |  |  |  | K.EFSPFGTITSAK.V |  |
|  |  |  |  | R.TVPQYKYAAGVR.N |  |
|  |  |  |  | R.IMWSQRDPSLR.K |  |
|  |  |  |  | R.QAHLTNQYMQR.M |  |
|  |  |  |  | R.QAHLTNQYMQR.M |  |
|  |  |  |  | R.QAHLTNQYMQR.M |  |
|  |  |  |  | K.SGVGNIFIKNLDK.S |  |
|  |  |  |  | R.QAHLTNQYMQR.M + Oxidation (M) |  |
|  |  |  |  | R.QAHLTNQYMQR.M + Oxidation (M) |  |
|  |  |  |  | R.KEFSPFGTITSAK.V |  |
|  |  |  |  | K.NLDDGIDDERLR.K |  |
|  |  |  |  | K.NLDDGIDDERLR.K |  |
|  |  |  |  | R.ITRYQGVNLYVK.N |  |
|  |  |  |  | K.VDEAVAVLQAHQAK.E |  |
|  |  |  |  | K.EAAQKAVNSATGVPTV.- |  |
|  |  |  |  | R.IVATKPLYVALAQR.K |  |
|  |  |  |  | K.GFGFVCFSSPEEATK.A |  |
|  |  |  |  | R.IVATKPLYVALAQRK.E |  |
|  |  |  |  | R.IVATKPLYVALAQRK.E |  |
|  |  |  |  | R.SKVDEAVAVLQAHQAK.E |  |
| 3303 | HSPA1A | Heat shock 70 kDa protein 1 | 70,009 | 7 | 163 |
|  |  |  |  | R.LSKEEIER.M |  |
|  |  |  |  | K.VQVSYKGETK.A |  |
|  |  |  |  | K.VQVSYKGETK.A |  |
|  |  |  |  | K.DAGVIAGLNVLR.I |  |
|  |  |  |  | K.NALESYAFNMK.S |  |
|  |  |  |  | K.NALESYAFNMK.S + Oxidation (M) |  |
|  |  |  |  | R.TTPSYVAFTDTER.L |  |
| 8087 | FXR1 | Fragile X mental retardation syndrome-related protein 1 | 69,678 | 1 | 42 |
|  |  |  |  | K.VIQEIVDKSGVVR.V |  |
| 10492 | SYNCRIP (HNRNPQ) | Heterogeneous nuclear ribonucleoprotein Q | 69,560 | 10 | 319 |
|  |  |  |  | K.AGPIWDLR.L |  |
|  |  |  |  | K.LYNNHEIR.S |  |
|  |  |  |  | K.AFSQFGKLER.V |  |
|  |  |  |  | R.LMMDPLTGLNR.G |  |
|  |  |  |  | R.LMMDPLTGLNR.G + Oxidation (M) |  |
|  |  |  |  | R.LMMDPLTGLNR.G + Oxidation (M) |  |
|  |  |  |  | R.TGYTLDVTTGQR.K |  |
|  |  |  |  | R.TGYTLDVTTGQRK.Y |  |
|  |  |  |  | R.NLANTVTEEILEK.A |  |
|  |  |  |  | R.DLFEDELVPLFEK.A |  |
| 10432 | RBM14 | RNA-binding protein 14 | 69,449 | 12 | 311 |
|  |  |  |  | R.INVELSTK.G |  |
|  |  |  |  | R.LSESQLSFR.R |  |
|  |  |  |  | R.LAELSDYRR.L |  |
|  |  |  |  | R.LPDAHSDYAR.Y |  |
|  |  |  |  | R.SPTKSSLDYR.R |  |
|  |  |  |  | R.AQPSASLGVGYR.T |  |
|  |  |  |  | R.INVELSTKGQK.K |  |
|  |  |  |  | R.YSGSYNDYLR.A |  |
|  |  |  |  | R.AQPSVSLGAPYR.G |  |
|  |  |  |  | R.RLPDAHSDYAR.Y |  |
|  |  |  |  | R.TQPMTAQAASYR.A |  |
|  |  |  |  | R.TQPMTAQAASYR.A + Oxidation (M) |  |
| 1655 | DDX5 (p68) | Probable ATP-dependent RNA helicase DDX5 | 69,105 | 20 | 397 |
|  |  |  |  | R.DRGFGAPR.F |  |
|  |  |  |  | K.LLQLVEDR.G |  |
|  |  |  |  | K.STCIYGGAPK.G + Propionamide (C) |  |
|  |  |  |  | K.GPQIRDLER.G |  |
|  |  |  |  | K.FGNPGEKLVK.K |  |
|  |  |  |  | K.FGNPGEKLVK.K |  |
|  |  |  |  | R.TAQEVETYR.R |  |
|  |  |  |  | K.IVDQIRPDR.Q |  |
|  |  |  |  | K.QVSDLISVLR.E |  |
|  |  |  |  | K.APILIATDVASR.G |  |
|  |  |  |  | R.LKSTCIYGGAPK.G |  |
|  |  |  |  | R.KIVDQIRPDR.Q |  |
|  |  |  |  | R.TAQEVETYRR.S |  |
|  |  |  |  | R.LKSTCIYGGAPK.G + Propionamide (C) |  |
|  |  |  |  | R.MLDMGFEPQIR.K |  |
|  |  |  |  | K.LLQLVEDRGSGR.S |  |
|  |  |  |  | K.LLQLVEDRGSGR.S |  |
|  |  |  |  | K.NFYQEHPDLAR.R |  |
|  |  |  |  | K.WNLDELPKFEK.N |  |
|  |  |  |  | K.NFYQEHPDLARR.T |  |
| 2130 | EWSR1 | RNA-binding protein EWS | 68,436 | 7 | 83 |
|  |  |  |  | K.LKVSLAR.K |  |
|  |  |  |  | R.GGPGGPGGPGGPMGR.M + Oxidation (M) |  |
|  |  |  |  | K.GDATVSYEDPPTAK.A |  |
|  |  |  |  | K.AAVEWFDGKDFQGSK.L |  |
|  |  |  |  | R.GMPPPLRGGPGGPGGPGGPMGR.M + Oxidation (M) |  |
|  |  |  |  | R.GMPPPLRGGPGGPGGPGGPMGR.M + 2 Oxidation (M) |  |
|  |  |  |  | R.QDHPSSMGVYGQESGGFSGPGENR.S + Oxidation (M) |  |
| 8880 | FUBP1 | Far upstream element-binding protein 1 | 67,518 | 1 | 44 |
|  |  |  |  | R.IGGNEGIDVPIPR.F |  |
| 51574 | LARP7 | La-related protein 7 | 66,857 | 9 | 131 |
|  |  |  |  | K.HIRFSEYD.- |  |
|  |  |  |  | K.IISTEPLPGRK.Q |  |
|  |  |  |  | K.TVKNKPIPALR.V |  |
|  |  |  |  | R.SSSEDAESLAPR.S |  |
|  |  |  |  | R.DRVEASSLPEVR.T |  |
|  |  |  |  | R.DRVEASSLPEVR.T |  |
|  |  |  |  | K.VNATGPQFVSGVIVK.I |  |
|  |  |  |  | K.AIEFLNNPPEEAPR.K |  |
|  |  |  |  | K.GFAFVEFETKEQAAK.A |  |
| 10644 | IGF2BP2 | Insulin-like growth factor 2 mRNA-binding protein 2 | 66,081 | 3 | 77 |
|  |  |  |  | K.IAPAEGPDVSER.M |  |
|  |  |  |  | R.MVIITGPPEAQFK.A |  |
|  |  |  |  | R.MVIITGPPEAQFK.A + Oxidation (M) |  |
| 253943 | YTHDF3 | YTH domain family protein 3 | 63,822 | 7 | 153 |
|  |  |  |  | R.VFIIK.S |  |
|  |  |  |  | R.WVAPR.N |  |
|  |  |  |  | K.IIATFK.H |  |
|  |  |  |  | K.IGGDLTAAVTK.T |  |
|  |  |  |  | R.DTQEVPLEK.A |  |
|  |  |  |  | K.VPGISSIEQGMTGLK.I |  |
|  |  |  |  | R.AITDGQAGFGNDTLSK.V |  |
| 10643 | IGF2BP3 | Insulin-like growth factor 2 mRNA-binding protein 3 | 63,681 | 4 | 115 |
|  |  |  |  | R.VPSFAAGR.V |  |
|  |  |  |  | K.ILAHNNFVGR.L |  |
|  |  |  |  | R.MVIITGPPEAQFK.A |  |
|  |  |  |  | R.MVIITGPPEAQFK.A + Oxidation (M) |  |
| 10642 | IGF2BP1 | Insulin-like growth factor 2 mRNA-binding protein 1 | 63,417 | 15 | 243 |
|  |  |  |  | R.FASASIK.I |  |
|  |  |  |  | K.MILEIMHK.E |  |
|  |  |  |  | K.MILEIMHK.E + Oxidation (M) |  |
|  |  |  |  | R.QGSPVAAGAPAK.Q |  |
|  |  |  |  | K.IAPPETPDSK.V |  |
|  |  |  |  | K.QQQVDIPLR.L |  |
|  |  |  |  | K.RLEIEHSVPK.K |  |
|  |  |  |  | K.IAPPETPDSKVR.M |  |
|  |  |  |  | K.IAPPETPDSKVR.M |  |
|  |  |  |  | R.MVIITGPPEAQFK.A |  |
|  |  |  |  | R.MVIITGPPEAQFK.A + Oxidation (M) |  |
|  |  |  |  | R.LLVPTQYVGAIIGK.E |  |
|  |  |  |  | K.LETHIRVPASAAGR.V |  |
|  |  |  |  | K.LETHIRVPASAAGR.V |  |
|  |  |  |  | R.DQTPDENDQVIVK.I |  |
| 51441 | YTHDF2 | YTH domain family protein 2 | 62,296 | 4 | 65 |
|  |  |  |  | R.VFIIK.S |  |
|  |  |  |  | R.WVAPR.N |  |
|  |  |  |  | R.DTQEVPLEK.A |  |
|  |  |  |  | K.LGSTEVASNVPK.V |  |
| 8939 | FUBP3 | Far upstream element-binding protein 3 | 61,602 | 10 | 279 |
|  |  |  |  | K.VGLVIGR.G |  |
|  |  |  |  | R.DGFGGLAAAR.G |  |
|  |  |  |  | R.GVPQQIEVAR.Q |  |
|  |  |  |  | K.AEGFVDALHR.V |  |
|  |  |  |  | R.LLGQIVDRCR.N |  |
|  |  |  |  | R.MGGGSIEVSVPR.F |  |
|  |  |  |  | R.MGGGSIEVSVPR.F + Oxidation (M) |  |
|  |  |  |  | R.GGETIKQLQER.T |  |
|  |  |  |  | R.ITGDAFKVQQAR.E |  |
|  |  |  |  | R.IQFKPDDGISPER.A |  |
| 3329 | HSPD1 | 60 kDa heat shock protein, mitochondrial | 61,016 | 2 | 59 |
|  |  |  |  | K.LSDGVAVLK.V |  |
|  |  |  |  | R.VTDALNATR.A |  |
| 9908 | G3BP2 | Ras GTPase-activating protein-binding protein 2 | 54,088 | 6 | 37 |
|  |  |  |  | R.GIVGGGMMR.D |  |
|  |  |  |  | R.GIVGGGMMR.D + Oxidation (M) |  |
|  |  |  |  | K.AFSWASVTSK.N |  |
|  |  |  |  | M.VMEKPSPLLVGR.E |  |
|  |  |  |  | R.VEAKPEVQSQPPR.V |  |
|  |  |  |  | R.ILIAKPIMFRGEVR.L |  |
| 280796 | FUS | RNA-binding protein FUS | 53,394 | 9 | 149 |
|  |  |  |  | K.VSFATR.R |  |
|  |  |  |  | K.QIGIIKTNK.K |  |
|  |  |  |  | K.TGQPMINLYTDR.E + Oxidation (M) |  |
|  |  |  |  | K.LKGEATVSFDDPPSAK.A |  |
|  |  |  |  | K.TGQPMINLYTDRETGK.L + Oxidation (M) |  |
|  |  |  |  | K.AAIDWFDGKEFSGNPIK.V |  |
|  |  |  |  | K.APKPDGPGGGPGGSHMGGNYGDDR.R |  |
|  |  |  |  | K.APKPDGPGGGPGGSHMGGNYGDDR.R |  |
|  |  |  |  | K.APKPDGPGGGPGGSHMGGNYGDDR.R + Oxidation (M) |  |
| 27316 | RBMX (HNRNPG) | Heterogeneous nuclear ribonucleoprotein G | 42,306 | 2 | 57 |
|  |  |  |  | R.SDLYSSGRDR.V |  |
|  |  |  |  | K.AIKVEQATKPSFESGR.R |  |
| 6895 | TARBP2 (TRBP) | RISC-loading complex subunit TARBP2 | 39,015 | 5 | 85 |
|  |  |  |  | R.RALQYLK.I |  |
|  |  |  |  | R.VHTVPLDAR.D |  |
|  |  |  |  | K.TPISLLQEYGTR.I |  |
|  |  |  |  | R.LPEYTVTQESGPAHR.K |  |
|  |  |  |  | R.DGNEVEPDDDHFSIGVGSR.L |  |
| 3178 | HNRNPA1 | Heterogeneous nuclear ribonucleoprotein A1 | 38,822 | 3 | 176 |
|  |  |  |  | K.VDGRVVEPK.R |  |
|  |  |  |  | R.SSGPYGGGGQYFAKPR.N |  |
|  |  |  |  | R.SSGPYGGGGQYFAKPR.N |  |
| 255022 | CALHM1 | Calcium homeostasis modulator protein 1 | 38,238 | 1 | 34 |
|  |  |  |  | K.SKYWSHYIDIER.K |  |
| 5093 | PCBP1 (HNRNPE1) | Poly(rC)-binding protein 1 | 37,474 | 3 | 41 |
|  |  |  |  | R.QGANINEIR.Q |  |
|  |  |  |  | K.IANPVEGSSGR.Q |  |
|  |  |  |  | R.INISEGNCPER.I |  |
| 3181 | HNRNPA2B1 | Heterogeneous nuclear ribonucleoproteins A2/B1 | 37,407 | 6 | 45 |
|  |  |  |  | R.GGNFGFGDSR.G |  |
|  |  |  |  | K.TLETVPLER.K |  |
|  |  |  |  | R.QEMQEVQSSR.S |  |
|  |  |  |  | R.SGRGGNFGFGDSR.G |  |
|  |  |  |  | K.ALSRQEMQEVQSSR.S |  |
|  |  |  |  | K.ALSRQEMQEVQSSR.S + Oxidation (M) |  |
| 6396 | SEC13 | Protein SEC13 homolog | 35,518 | 2 | 54 |
|  |  |  |  | R.SVKIFDVR.N |  |
|  |  |  |  | R.NGGQILIADLR.G |  |
| 3183 | HNRNPC | Heterogeneous nuclear ribonucleoproteins C1/C2 | 33,650 | 1 | 40 |
|  |  |  |  | R.VPPPPPIAR.A |  |
| 292 | SLC25A5 | ADP/ATP translocase 2 | 32,874 | 7 | 68 |
|  |  |  |  | K.TAVAPIER.V |  |
|  |  |  |  | K.GAWSNVLR.G |  |
|  |  |  |  | K.TAVAPIERVK.L |  |
|  |  |  |  | R.LAADVGKAGAER.E |  |
|  |  |  |  | R.AAYFGIYDTAK.G |  |
|  |  |  |  | K.DFLAGGVAAAISK.T |  |
|  |  |  |  | R.EFRGLGDCLVK.I |  |
| 293 | SLC25A6 | ADP/ATP translocase | 32,845 | 5 | 43 |
|  |  |  |  | K.TAVAPIER.V |  |
|  |  |  |  | K.GAWSNVLR.G |  |
|  |  |  |  | K.TAVAPIERVK.L |  |
|  |  |  |  | R.GLGDCLVKITK.S |  |
|  |  |  |  | R.EFRGLGDCLVK.I |  |
| 6187 | RPS2 | 40S ribosomal protein S2 | 31,305 | 3 | 40 |
|  |  |  |  | K.ATFDAISK.T |  |
|  |  |  |  | R.GTGIVSAPVPK.K |  |
|  |  |  |  | R.VSVQRTQAPAVATT.- |  |
| 6189 | RPS3A | 40S ribosomal protein S3a | 29,926 | 10 | 131 |
|  |  |  |  | K.MMEIMTR.E |  |
|  |  |  |  | K.APAMFNIR.N |  |
|  |  |  |  | K.MMEIMTR.E + Oxidation (M) |  |
|  |  |  |  | R.NIGKTLVTR.T |  |
|  |  |  |  | K.LITEDVQGK.N |  |
|  |  |  |  | R.TQGTKIASDGLK.G |  |
|  |  |  |  | R.TQGTKIASDGLK.G |  |
|  |  |  |  | K.LMELHGEGSSSGK.A |  |
|  |  |  |  | R.EVQTNDLKEVVNK.L |  |
|  |  |  |  | K.VERADGYEPPVQESV.- |  |
| 6191 | RPS4X | 40S ribosomal protein S4, X isoform | 29,579 | 3 | 46 |
|  |  |  |  | R.IGVITNR.E |  |
|  |  |  |  | R.LSNIFVIGK.G |  |
|  |  |  |  | R.IGVITNRER.H |  |
| 6132 | RPL8 | 60S ribosomal protein L8 | 28,007 | 3 | 50 |
|  |  |  |  | R.KVGLIAAR.R |  |
|  |  |  |  | R.AVVGVVAGGGR.I |  |
|  |  |  |  | R.ASGNYATVISHNPETK.K |  |
| 6188 | RPS3 | 40S ribosomal protein S3 | 26,671 | 3 | 58 |
|  |  |  |  | R.ELAEDGYSGVEVR.V |  |
|  |  |  |  | K.DEILPTTPISEQK.G |  |
|  |  |  |  | K.GGKPEPPAMPQPVPTA.- |  |
